# Supplementary material for: Curcumin-like Compound Inhibits Proliferation of Adenocarcinoma Cells by Inducing Cell Cycle Arrest and Senescence
Source: Pharmaceuticals (Basel). 2025 Jun 18;18(6):914. doi: 10.3390/ph18060914 (PMC12196036; doi:10.3390/ph18060914)
Supplement: Supplementary file 1 [file pharmaceuticals-18-00914-s001.zip › pharmaceuticals-3590466-Suplementary.pdf]

Article

Curcumin-like Compound Inhibits Proliferation of Adenocarcinoma Cells by Inducing Cell Cycle Arrest and Senescence

Rafael Fonseca[1], Yasmin dos Santos Louzano[1], Cindy Juliet Cristancho Ortiz[2], Matheus de Freitas Silva[2], [1], Maria Luiza Vieira Felix[1], Guilherme Álvaro Ferreira-Silva[1], Ester Siqueira Caixeta[1], Bruno Zavan[1], Claudio Viegas Jr.[2,] *, and Marisa Ionta [1,*]

1 Laboratório de Avaliação de Protótipos Antitumorais (LAPAN), Instituto de Ciências Biomédicas, Universidade Federal de Alfenas (UNIFAL-MG), Alfenas 37130-001, Brazil; rafael.miranda@sou.unifal-mg.edu.br (R.F.); yasmin.louzano@sou.unifal-mg.edu.br (Y.S.L.); maria.felix@sou.unifal-mg.edu.br (M.L.V.F.); brunozavan@yahoo.com.br (B.Z.); ester.caixeta@unifal-mg.edu.br (E.S.C)

2 Laboratory of Research in Medicinal Chemistry, Institute of Chemistry, Federal University of Alfenas, Alfenas, Minas Gerais, Brazil; cjcristanchoo@unal.edu.co (C.J.C.O.); defreitassilva.matheus@gmail.com (M.F.S.),

* Correspondence: marisa.ionta@unifal-mg.edu.br (M.I.); claudio.viegas@unifal-mg.edu.br (C.V.J.)

Supplementary material

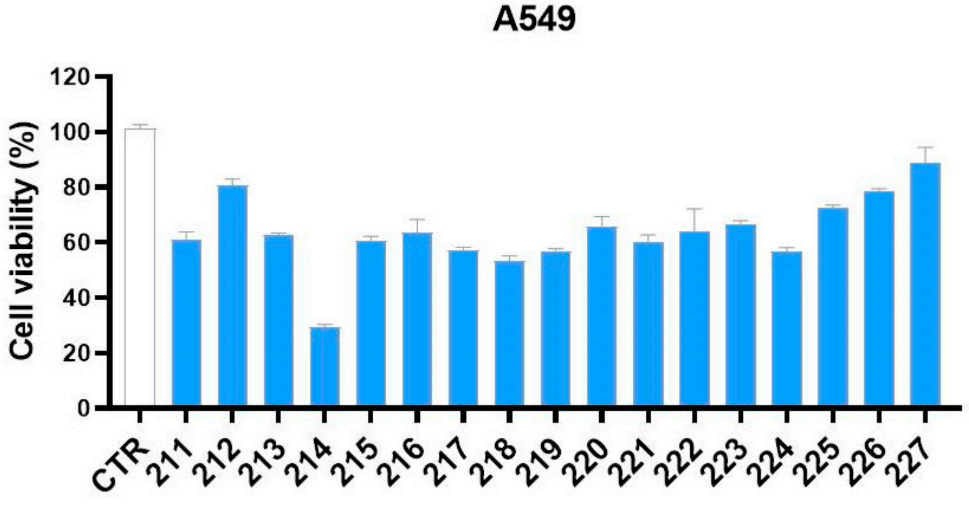

Figure S1. Cell viability was determined after 48 hours of treatment with different substances on A549 cells.

Table S1. Primers used for relative quantification in real-time RT-qPCR

Gene	Sequence	Reference
CDKN1A	F 5’- CCATAGCCTCTACTGCCACCATC-3’<br>R 5’- GTCCAGCGACCTTCCTCATCCA-3’	NM_001291549.1

CCNE2	F 5’- GGCTATGCTGGAGGAAGTAAAT-3’	NM_057749.2
	R 5’- GCTCTTCGGTGGTGTCATAAT-3’	
CCND1	F 5’- GGGTTGTGCTACAGATGATAGAG-3’	NM_053056.2
	R 5’- AGACGCCTCCTTTGTGTTAAT-3’	
FOXM1	F 5’- TGCCCAGCAGTCTCTTACCT-3’	NM_001243089.1
	R 5’- CTACCCACCTTCTGGCAGTC-3’	
IL8	F 5’- CTTGGCAGCCTTCCTGATTT -3’	NM_000584.4
	R 5’- GGGTGGAAAGGTTTGGAGTATG -3’	
LMNB1	F 5’- GAGAGAGAGATGGCGGAAATAAG -3’	NM_005573.4
	R 5’- TTTCCATGTCCAGGGCTAAC -3’	
TIMP1	F:5’- ATGGACTCTTGCACATCACTAC -3’	NM_003254.3
	R:5’- GGGATGGATAAACAGGGAAACA -3’	
TIMP2	F:5’- AGGGCCTGAGAAGGATATAGAG -3’	NM_003255.5
	R:5’- GGCCTTTCCTGCAATGAGATA -3’	
BCL2	F:5’- CAGAAGTCTGGGAATCGATCTG -3’	NM_000657.2
	R:5’- AATCTTCAGCACTCTCCAGTTATAG -3’	
BAX	F:5’- TTCCTTACGTGTCTGATCAATCC -3’	NM_004324.3
	R:5’- GGGCAGAAGGCACTAATCAA -3’	
ACTB	F 5’- AGAGCTACGAGCTGCCTGAC-3’	NM_001101.3
	R 5’- AGCACTGTGTTGGCGTACAG-3’	

F = forward primer; R = reverse primer

Table S2: List of antibodies used in western blot analyses

Antibody	Dilution	Source	Code
c-Myc	1:1,000	Cell Signaling	#5605
Cyclin B1	1:1,000	Cell Signaling	#12231
Cyclin D1	1:1,000	Cell Signaling	#55506
p21	1:1,000	Cell Signaling	#2947
α-Tubulin	1:1,000	Cell Signaling	#3873
β-Actin	1:10,000	Sigma	A3854
